# Integrating network pharmacology and bioinformatics to explore the mechanism of Xiaojian Zhongtang in treating major depressive disorder: An observational study

**DOI:** 10.1097/MD.0000000000039726

**Published:** 2024-09-20

**Authors:** Huaning Jiang, Jian Zhang, Quan Li, Yanyan Zhou

**Affiliations:** a School of Basic Medicine, Heilongjiang University of Traditional Chinese Medicine, Harbin, China.

**Keywords:** bioinformatics, major depressive disorder, molecular mechanisms, network pharmacology, Xiaojian Zhongtang

## Abstract

Major depressive disorder (MDD) is a common mental illness. The traditional Chinese medicine compound Xiaojian Zhongtang (XJZT) has a good therapeutic effect on MDD, but the specific mechanism is not clear. The aim of this study is to explore the molecular mechanism of XJZT in the treatment of MDD through network pharmacology and bioinformatics. The traditional Chinese medicine system pharmacology database was used to screen the chemical components and targets of XJZT, while the online Mendelian inheritance in man, DisGeNET, Genecards, and therapeutic target database databases were used to collect MDD targets and identify the intersection targets of XJZT and MDD. A “drugs-components-targets” network was constructed using the Cytoscape platform, and the STRING was used for protein-protein interaction analysis of intersecting targets. Gene Ontology and Kyoto encyclopedia of genes and genomes analysis of intersecting targets was performed using the DAVID database. Obtain serum and brain transcriptome datasets of MDD from the gene expression omnibus database, and perform differentially expressed genes, weighted gene co-expression network analysis, gene set enrichment analysis, and receiver operating characteristic analysis. A total of 127 chemical components and 767 targets were obtained from XJZT, among which quercetin, kaempferol, and maltose are the core chemical components, and 1728 MDD targets were screened out, with 77 intersecting targets between XJZT and MDD. These targets mainly involve AGE-RAGE signaling pathway in diabetic complexes, epidermal growth factor receptor tyrosine kinase inhibitor resistance, and HIF-1 signaling pathway, and these core targets have strong binding activity with core components. In addition, 1166 differentially expressed genes were identified in the MDD serum transcriptome dataset, and weighted gene co-expression network analysis identified the most relevant gene modules (1269 genes), among which RAC-alpha serine/threonine-protein kinase (AKT1), D(4) dopamine receptor (DRD4), and kynurenine 3-monooxygenase (KMO) were target genes for the treatment of MDD with XJZT, these 3 genes are mainly related to the ubiquitin-mediated proteolysis, arachidonic acid (AA) metabolism, and Huntington disease pathways, and the expression of AKT1, DRD4, and KMO was also found in the MDD brain transcriptome dataset, which is significantly correlated with the occurrence of MDD. We have identified 3 key targets for XJZT treatment of MDD, including AKT1, KMO, and DRD4, and they can be regulated by the key components of XJZT, including quercetin, maltose, and kaempferol. This provides valuable insights for the early clinical diagnosis and development of therapeutic drugs for MDD.

## 1. Introduction

Major depressive disorder (MDD) is a mental illness characterized by sustained low mood and loss of pleasure, and is also considered one of the largest causes of disability worldwide.^[1]^ But, there are many other accompanying symptoms of MDD, including reduced diet, reduced sleep time, decreased memory, and delayed mental activity. As these symptoms persist and the effectiveness of antidepressants gradually decreases, it ultimately leads to patients in a chronic and refractory state of depression.^[2,3]^ However, in clinical practice, there are often physical and cognitive symptoms related to anxiety, which are difficult to distinguish from normal emotional expression, making it one of the biggest obstacles to early diagnosis of MDD. Therefore, MDD does not seem to be a standalone disease, but rather a combination of “diverse” symptoms.^[4]^ Studies have shown that approximately 350 million people worldwide are prone to depression, with a lifetime prevalence of about 15%. With the acceleration of society and the pace of life, MDD will be one of the important contributors to the global disease burden.^[5]^

The causes of MDD are very complex, and the pathogenesis is not yet clear. At present, it is widely believed that the pathogenesis of MDD is related to abnormalities in 2 neural circuits. One is the implicit emotion regulation circuit mediated by 5-hydroxytryptamine (5-HT), which centers on the medial prefrontal cortex and amygdala, including the anterior cingulate cortex, dorsal prefrontal cortex, and hippocampus.^[6]^ Another is the dopamine-mediated reward neural circuit, centered around the medial prefrontal cortex, ventral striatum, and nucleus accumbens.^[7]^ The abnormalities of 2 neural circuits in MDD patients include multiple levels of abnormalities such as gray matter volume, resting function connectivity, and brain metabolism. In addition, dysfunction of the hypothalamic pituitary adrenal axis is also an important pathway leading to the occurrence of MDD. Chronic stress stimulation keeps the hypothalamic pituitary adrenal axis in an activated state for a long time, and the levels of adrenocorticotropic hormone and glucocorticoid secretion continue to rise, leading to the production of inflammatory cytokines (interleukin-6 [IL-6], tumor necrosis factor [TNF]-α) in cells, and the release of neurotransmitters, such as gamma-aminobutyric acid and brain-derived neurotrophic factor (BDNF) decreases.^[8–11]^ It is worth mentioning that the cholinergic system is also related to the pathogenesis of depression, as the muscarinic cholinergic system is overly active or reactive in depression.^[12]^

The treatment of MDD currently mainly relies on antidepressants, with commonly used drugs being selective serotonin reuptake inhibitors (SSRIs), serotonin and norepinephrine reuptake inhibitors, supplemented by psychotherapy, physical therapy, and so on.^[13]^ The international guidelines currently recommend SSRI drugs as the first-line treatment for most MDD patients. Common SSRI drugs include sertraline, escitalopram, fluoxetine, paroxetine, etc.^[14]^ SSRI drugs can enhance synaptic plasticity, but the response to drug therapy varies greatly among individuals.^[15]^ Serotonin and norepinephrine reuptake inhibitor is a dual channel antidepressant drug, represented by venlafaxine and duloxetine, which inhibits the reuptake of 5-HT and norepinephrine by the presynaptic membrane in the synaptic gap.^[16]^ However, it becomes increasingly difficult to achieve symptom relief after each treatment failure, and the relief rate decreases with the frequency of subsequent antidepressant treatment.^[17]^

Traditional Chinese medicine theory suggests that the physiological functions of the organs are related to emotional activities, and they can interact with each other. The spleen corresponds to worry and anxiety, so excessive anxiety can affect the function of the spleen. In fact, MDD patients may also exhibit some digestive dysfunction in clinical practice, including decreased appetite, pain, and indigestion. Xiaojian Zhongtang (XJZT) is derived from the classic Chinese medicine work “Treatise on Cold Damage and Miscellaneous Diseases.” It is composed of *Cinnamomi Ramulus* (Chinese name: Gui Zhi), *Paeoniae Radix Alba* (Chinese name: Bai Shao), *Zingiber Officinale Roscoe* (Chinese name: Sheng Jiang), *Jujubae Fructus* (Chinese name: Da Zao), maltose (Chinese name: Yi Tang), and *licorice* (Chinese name: Gan Cao), and has the function of strengthening the spleen and stomach, warming yang and dispelling cold.^[18]^ Modern pharmacological studies have shown that drugs that makeup XJZT also have antidepressant effects. Bai Shao extract can reduce the expression of L-tryptophan (TRP) catabolizing enzyme tryptophan 2,3-dioxygenase (TDO) in the liver of stress-induced depression-like mice.^[19]^ In addition, extracts of Sheng Jiang and Gui Zhi can also reduce the expression of P53 and glutathione reductase genes in the brain of depression model rats, and upregulate the expression of glutathione peroxidase 1 gene, improving depression symptoms in rats.^[20,21]^ But the specific mechanism of XJZT antidepressant effect is not yet clear.

**Figure 1.**
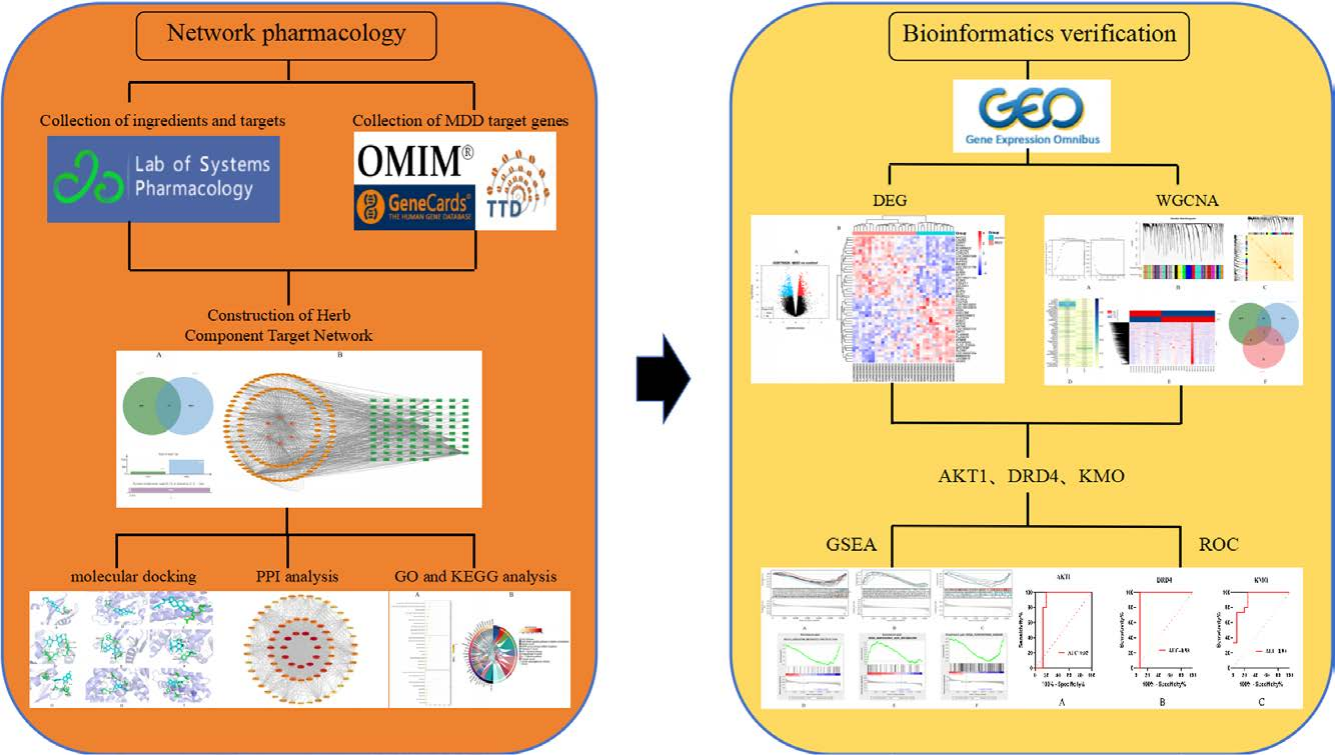
Workflow diagram of this study. AKT1 = RAC-alpha serine/threonine-protein kinase, DRD4 = dopamine D4 receptor, GO = gene ontology, GSEA = gene set enrichment analysis, KEGG = Kyoto encyclopedia of genes and genomes, KMO = kynurenine 3-monooxygenase, MDD = major depressive disorder, OMIM = online Mendelian inheritance in man, PPI = protein-protein interaction, ROC = receiver operating characteristic.

Therefore, this study utilized network pharmacology to screen potential target genes and mechanisms of action of XJZT in the treatment of MDD, and validated the molecular and biological mechanisms of XJZT intervention in MDD using bioinformatics, in order to provide reference and guidance for traditional Chinese medicine treatment of MDD (Fig. 1).

## 2. Materials and methods

### 2.1. Chemical composition and target screening of XJZT

In traditional Chinese medicine system pharmacology (TCMSP) (https://old.tcmsp-e.com/), HERB database (http://herb.ac.cn/) obtain the chemical components of XJZT, and set the screening conditions as oral bioavailability ≥30% and drug-like properties (DL) ≥0.18. Put the obtained chemical components in the PharMapper (http://www.lilab-ecust.cn/pharmmapper/) collect potential targets on TCMSP, save the mol.2 format file of the structural formula of ingredients in the TCMSP database, and upload it to the PharmaMapper website. Set the filtering conditions “Human Protein Targets Only” and “Normalized Fit Score ≥ 0.8” in the task submission options for screening. Through UniProt (https://www.uniprot.org/) convert the database to standard format gene names and save them. This study does not involve human or animal experiments, no ethical approval is required.

### 2.2. MDD target screening

Use “major depression disorder” as the search term in the online Mendelian inheritance in man database (http://www.omim.org), therapeutic target database (https://db.idrblab.net/ttd/), GeneCards database (https://www.genecards.org), DisGeNET database (https://www.disgenet.org) collect target genes related to MDD in Venny 2.1.0 (https://bioinfogp.cnb.csic.es/tools/venny/index.html) screen out the intersection targets of MDD and XJZT and draw a Venn diagram.

### 2.3. Protein-protein interaction analysis

Upload the intersection target genes to the STRING database (https://cn.string-db.org/), set the biological species option to “Homo sapien,” set the minimum interaction threshold to “Highest confidence > 0.9,” set other parameters to default values, and download the tsv format file. Use CytoHubba and MCODE plugins to screen for core and Hub target gene sets, and construct a visual protein-protein interaction (PPI) network.

### 2.4. Construction of the “drugs-components-targets” network

Import the active ingredients of XJZT and its intersection targets with MDD into Cytoscape 3.9.1, construct a “drugs-components-targets” network, and screen the core chemical components based on in-degree values.

### 2.5. Enrichment analysis

Using the DAVID database (https://david.ncifcrf.gov/tools.jsp) perform gene ontology (GO) analysis and Kyoto encyclopedia of genes and genomes (KEGG) analysis on intersection targets, with *P* < 0.05 as the limiting condition, screen for cellular components, biological processes, molecular functions, and enrichment pathways, and use R language for visual analysis.

### 2.6. Molecular docking

Using AutoDock Vina (http://vina.scripps.edu/) docking chemical components with disease targets. In the protein data bank database (https://www.rcsb.org/). Obtain the target protein and preprocess the obtained crystal structure in the PubChem database (https://pubchem.ncbi.nlm.nih.gov/) download ligand structure. Finally, molecular docking was performed between these target structures and the active ingredient structures using Pyrx (https://pyrx.sourceforge.io/) and internal vina docking using Pymol (https://pymol.org/2/) visualize and analyze it.

### 2.7. Gene expression omnibus dataset acquisition for MDD

Using “major depressive disorder” as the search term, we screened the gene expression dataset in the gene expression omnibus (GEO) database (http://www.ncbi.nlm.nih.gov/geo/) and ultimately obtained the blood transcriptome dataset GSE76826 (including 12 normal cases and 20 MDD cases) and the brain transcriptome dataset GSE54568 (including 15 normal cases and 15 MDD cases).

### 2.8. Differential gene analysis

Perform differentially expressed gene (DEG) analysis on the GSE76826 dataset using the “GEO2R” function in the GEO database, with the following filtering criteria: adj *P* val <0.05, *P* value <0.05, 0.5 < log2FC <−0.5, and select the first 50 DEG to draw a heat map.

### 2.9. Weighted gene co-expression network analysis

Convert the probe names in the series expression matrices of the GSE76826 dataset to gene names, eliminate abnormal and non-gene named data, and then perform weighted gene co-expression network analysis (WGCNA) on the series expression matrices of the 2 datasets using R software to screen out the core module gene set.

### 2.10. Gene set enrichment analysis

Convert the probe names in the series expression matrix of the GSE76826 dataset to gene names, remove abnormal and non-gene-named data, and then upload them to the gene set enrichment analysis (GSEA) software (version 3.0) for GSEA analysis of the common genes of the core module genes and DEGs. Based on the gene expression profile and phenotype grouping, set the minimum gene set to 5, the maximum gene set to 500, 1000 resampling, *P* value < 0.05, FDR of <0.25.

### 2.11. Receiver operating characteristic analysis

We conducted receiver operating characteristic (ROC) analysis on the core target genes of XJZT in treating MDD using the R software package pROC (version 1.17.0.1) to obtain area under ROC curve (AUC). Specifically, we obtained the core target genes expression of XJZT for MDD treatment in the GSE54568 dataset, conducted correlation analysis using the ROC function of pROC, and evaluated AUC and confidence intervals using the ci function of pROC to obtain the final AUC results.

## 3. Results

### 3.1. Chemical composition and MDD target screening results of XJZT

**Figure 2.**
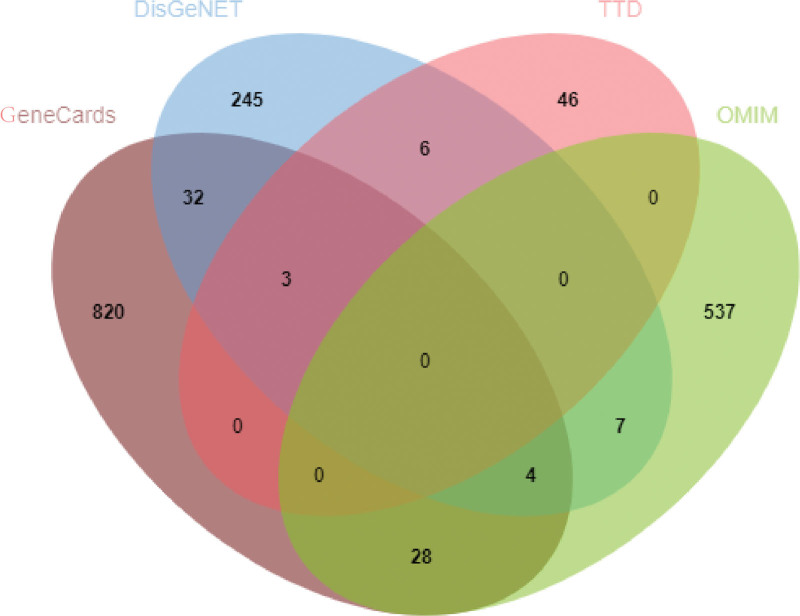
Venn plot of MDD target genes. 887, 297, 55, and 576 MDD target genes were screened in GeneCards, DisGeNET, TTD, and OMIM databases, respectively. MDD = major depressive disorder, OMIM = online Mendelian inheritance in man, TDD = therapeutic target database.

Through searching in TCMSP and HERB databases, we obtained a total of 127 chemical components in XJZT, including 7 components belonging to Gui Zhi, 13 components belonging to Bai Shao, 5 components belonging to Sheng Jiang, 5 components belonging to Da Zao, 93 components belonging to Gan Cao, and 4 components belonging to Yi Tang. In addition, there are a total of 767 potential targets for these chemical components, among which Gui Zhi, Sheng Jiang, Bai Shao, Da Zao, Yi Tang, and Gan Cao correspond to 52, 56, 87, 213, 120, and 239 targets, respectively. Next, we searched for disease-target genes related to MDD in multiple databases. The results showed that we found 887, 297, 55, and 576 MDD-related targets in GeneCards, DisGeNET, therapeutic target database, and Mendelian inheritance in man databases, respectively (Fig. 2).

### 3.2. Construction results of “drugs-components-targets” network

**Figure 3.**
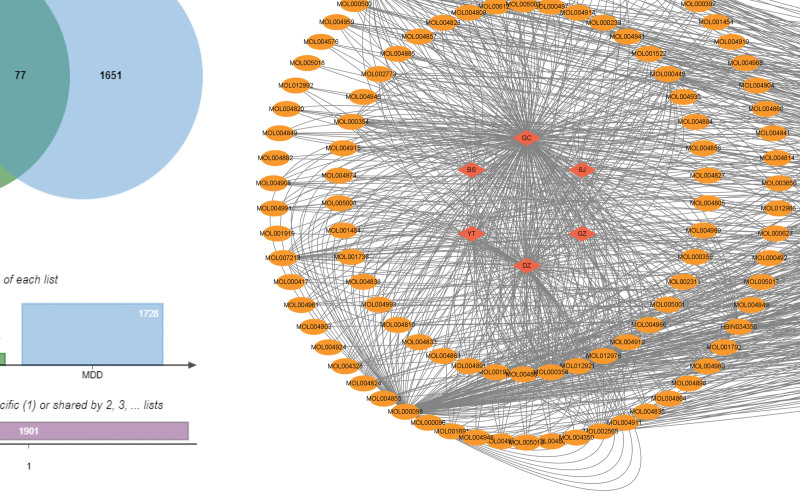
The diagram of Venn and network. (A) The diagram of Venn. After removing duplicate targets, XJZT ultimately obtained 327 targets, while MDD ultimately obtained 1728 targets, with 77 targets shared by XJZT and MDD. (B) The diagram of “drugs-components-targets” network. Pink represents the drugs that makeup XJZT, yellow represents the chemical composition of the drugs, and green represents the targets of XJZT for treating MDD, and quercetin, beta-sitosterol, maltose, stigmasterol, kaempferol, medicarpin, nuciferin, steroline, isorhamnetin, and stepharine are the core components. MDD = major depressive disorder, XJZT = Xiaojian Zhongtang.

In order to further identify the potential therapeutic targets of XJZT for MDD, we constructed a “drugs-components-targets” network. Our research results show that XJZT and MDD have a total of 77 intersecting targets, which may be important genes for XJZT to exert therapeutic effects on MDD (Fig. 3A and B). We selected the top 10 chemical components based on in-degree values as the core chemical components for XJZT intervention in MDD, including quercetin, beta-sitosterol, maltose, stigmasterol, kaempferol, medicarpin, nuciferin, steroline, isorhamnetin, and stepharine (Table 1).

**Table 1:** Core chemical composition of XJZT.

ID	Compound	In-degree	Betweenness centrality	Closeness centrality
MOL000098	quercetin	71	0.094934805	1
MOL000358	beta-sitosterol	34	0.083796627	1
HBIN034356	maltose	22	0.049968374	1
MOL000449	stigmasterol	21	0.0355552	1
MOL000422	kaempferol	19	0.032130392	1
MOL002565	medicarpin	14	0.00205076	1
MOL007213	nuciferin	12	0.007241704	1
MOL000627	stepholidine	10	0.007683983	1
MOL000354	isorhamnetin	10	0.00039942	1
MOL012921	stepharine	9	0.005302483	1

### 3.3. PPI analysis results

**Figure 4.**
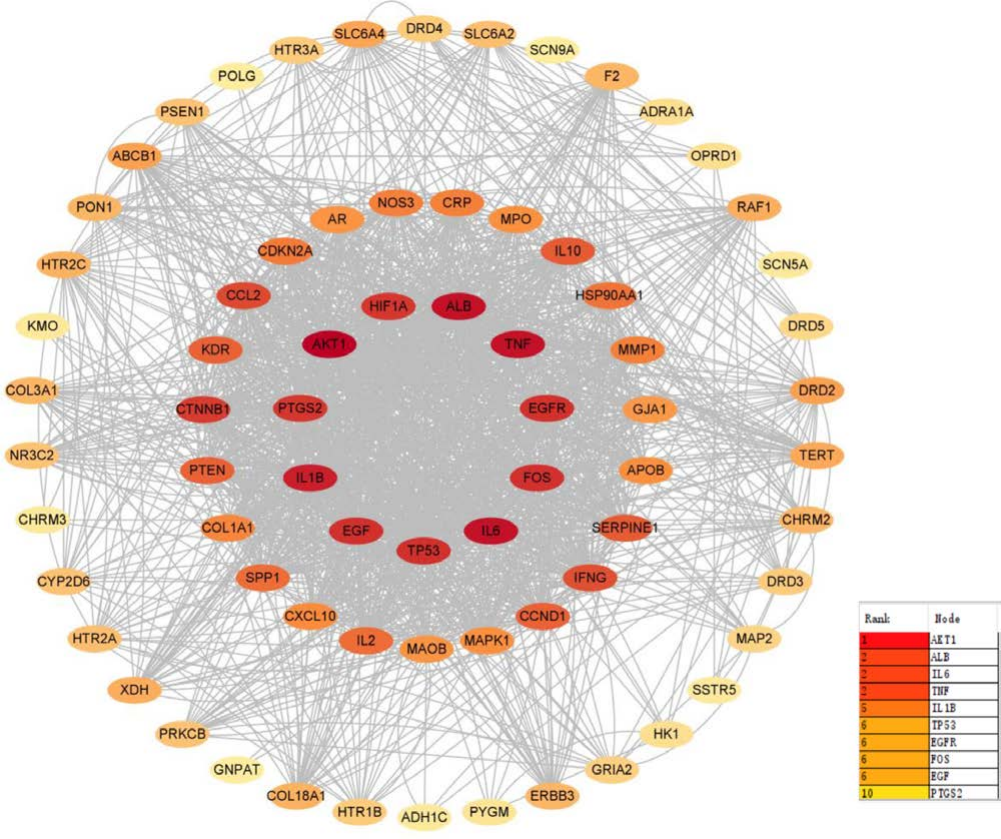
The diagram of PPI analysis. Seventy-seven target genes for XJZT treatment of MDD were uploaded to the STRING platform for analysis, the redder the color, the closer the interaction with other target genes. MDD = major depressive disorder, PPI = protein-protein interaction, XJZT = Xiaojian Zhongtang.

**Figure 5.**
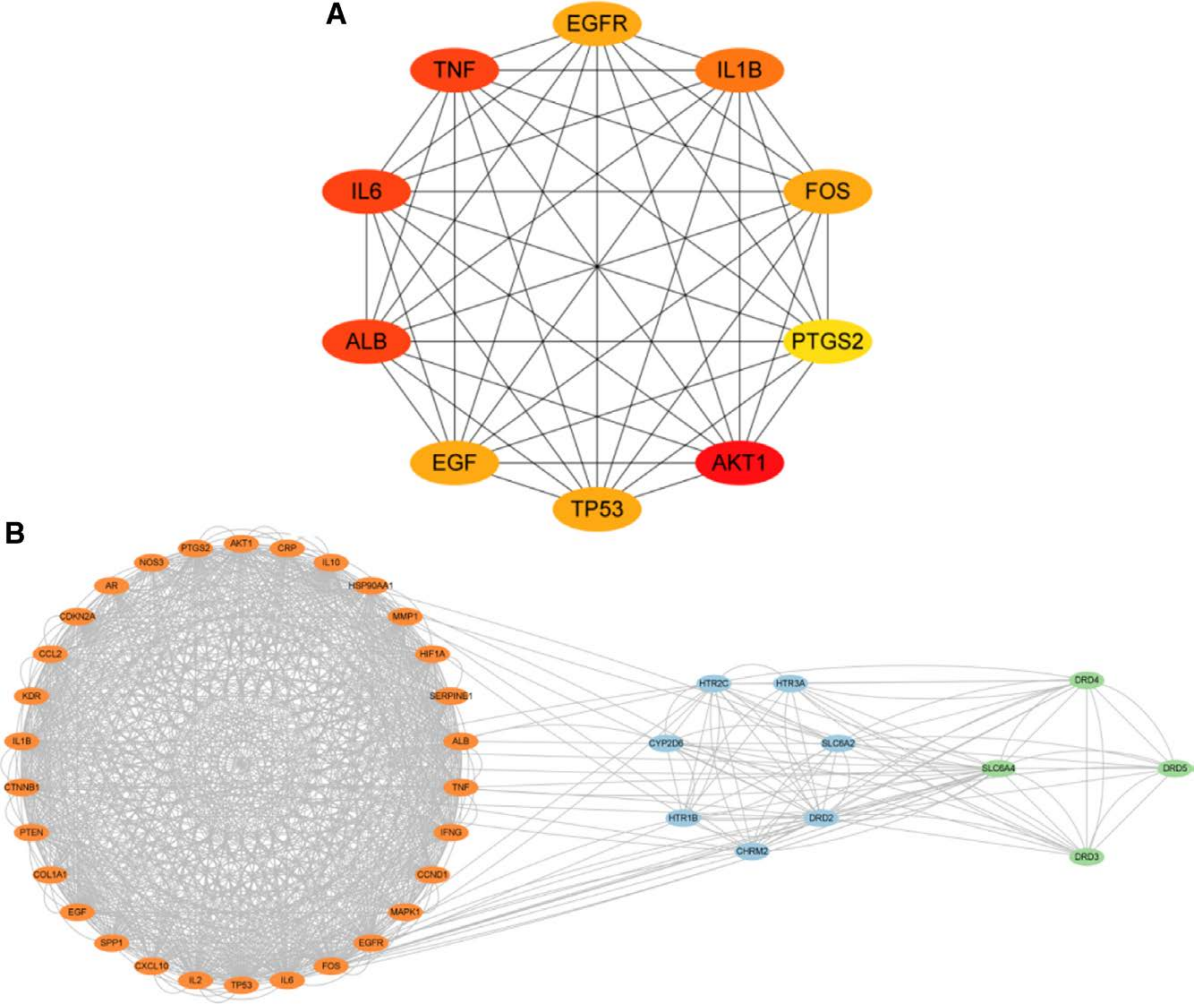
The diagram of mcode and cytohubba. (A) The diagram of cytohubba. In PPI analysis, the top 10 genes were selected based on their in-degree values and their interaction networks were displayed, including AKT1, ALB, IL-6, TNF, IL-1β, TP53, EGFR, FOS, EGF, and PTGS2 (B). The diagram of mcode. In PPI analysis, the mcode plugin was used to display the clustering of gene modules. A total of 3 gene modules were identified, with the orange gene module containing 30 target genes, the blue gene module containing 7 target genes, and the green gene module containing 4 target genes. AKT1 = RAC-alpha serine/threonine-protein kinase, ALB = albumin, EGF = epidermal growth factor, EGFR = epidermal growth factor receptor, FOS = protein c-fos, IL-1β = interleukin-1 beta, IL-6 = interleukin-6, PTGS2 = prostaglandin G/H synthase 2, TNF = tumor necrosis factor, TP53 = tumor protein 53.

After uploading the intersection target genes to the STRING platform, a total of 77 nodes and 338 edges were obtained (Fig. 4). Subsequently, the TSV file was imported into Cytoscape 3.9.1 for visual analysis. Using the MCODE plugin, 3 gene modules were identified, with the orange module being the largest, containing 30 target genes and 770 edges, the blue module containing 7 genes and 32 edges, the green module containing 4 genes and 12 edges (Fig. 5B). Using the CytoHubba plugin, the top 10 core target genes were screened, including RAC-alpha serine/threonine-protein kinase (AKT1), albumin, IL-6, TNF, interleukin-1 beta (IL-1β), tumor protein 53 (TP53), epidermal growth factor receptor (EGFR), protein c-fos, epidermal growth factor (EGF), and prostaglandin G/H synthase 2 (PTGS2) (Table 2, Fig. 5A).

**Table 2:** Core target genes of XJZT.

Gene	In-degree	Betweenness centrality	Closeness centrality
AKT1	47	2.442224545	0.724489796
ALB	44	1.415815703	0.696078431
IL-6	44	1.540221848	0.71
TNF	44	1.394466917	0.702970297
IL-1β	42	1.462639267	0.696078431
TP53	39	1.989690558	0.63963964
EGFR	39	0.981550186	0.663551402
FOS	39	2.644643521	0.676190476
EGF	39	0.527744105	0.633928571
PTGS2	38	1.39445536	0.669811321

AKT1 = RAC-alpha serine/threonine-protein kinase, ALB = albumin, EGF = epidermal growth factor, EGFR = epidermal growth factor receptor, FOS = protein c-fos, IL-1β = interleukin-1 beta, IL-6 = interleukin-6, PTGS2 = prostaglandin G/H synthase 2, TNF = tumor necrosis factor, TP53 = tumor protein 53.

### 3.4. GO and KEGG analysis results

**Figure 6.**
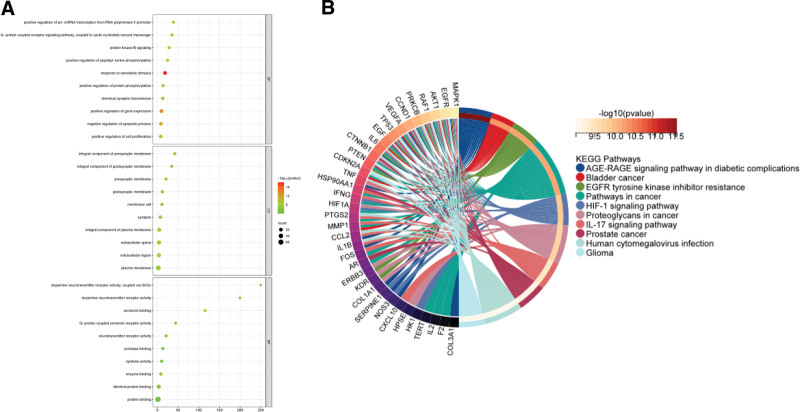
GO and KEGG analysis. (A) The GO analysis. The larger the point, the more target genes it contains, and the redder the color, the higher the significance. (B) The KEGG analysis. According to *P* values, the top 10 pathways are labeled in the graph. GO = gene ontology, KEGG = Kyoto encyclopedia of genes and genomes.

Import intersection target genes into DAVID data for GO analysis and KEGG. GO analysis resulted in a total of 2028 entries, of which 438 entries were related to biological processes, mainly involving response to xenobiotic stimuli, positive regulation of gene expression, negative regulation of apoptotic processes, etc. There are a total of 66 entries in cellular composition, mainly involving integral component of presynaptic membrane, extracellular space, and integral component of plasma membrane. Molecular function has a total of 65 entries, mainly involving serotonin binding, enzyme binding, G-protein coupled serotonin receptor activity (Fig. 6A). KEGG analysis identified a total of 147 pathways, mainly AGE-RAGE signaling pathway in radial composites, bladder cancer, EGFR tyrosine kinase inhibitor resistance, pathways in cancer, and HIF-1 signaling pathway (Fig. 6B).

### 3.5. Molecular docking results

**Figure 7.**
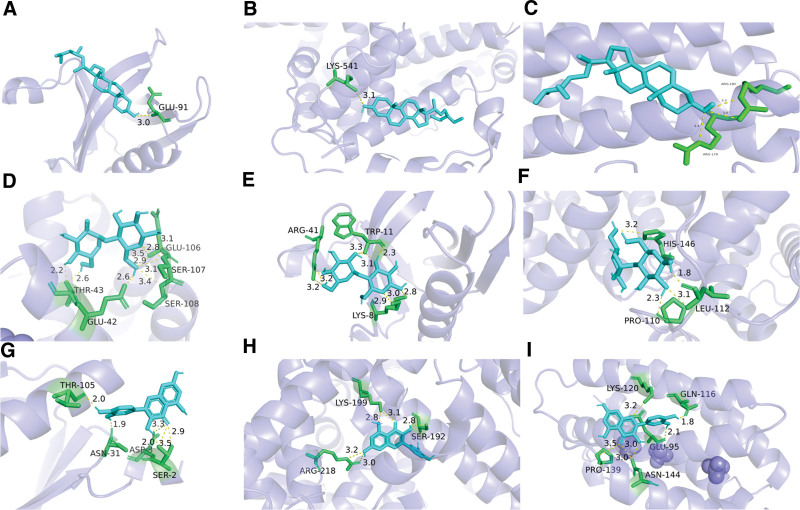
The diagram of molecular docking. (A) docking between beta-sitosterol and AKT1, binding energy is −6.9 kcal·mol^−1^; (B) docking between beta-sitosterol and ALB, binding energy is −8.5 kcal·mol^−1^; (C) docking between beta-sitosterol and IL-6, binding energy is −7.6 kcal·mol^−1^; (D) docking between maltose and IL-6, binding energy is −6.0 kcal·mol^−1^; (E) docking between maltose and AKT1, binding energy is −5.4 kcal·mol^−1^; (F) docking between maltose and ALB, binding energy is −5.6 kcal·mol^−1^; (G) docking between quercetin and AKT1, binding energy is −6.5kcal·mol^−1^; (H) docking between quercetin and ALB, binding energy is −7.9 kcal·mol^−1^; (I) docking between quercetin and IL-6, binding energy is −6.5 kcal·mol^−1^. AKT1 = RAC-alpha serine/threonine-protein kinase, ALB = albumin, IL-6 = interleukin-6.

In order to further verify the therapeutic effect of XJZT on MDD, we docked 10 core chemical components with 10 core target proteins, and the binding stability between ligands and receptors was negatively correlated with the binding energy value. Generally, when the binding energy is <−5.0 kcal·mol^−1^, it indicates good binding between small molecules of traditional Chinese medicine and target proteins; <−7.0 kcal·mol^−1^, it exhibits strong binding activity. The results showed that the binding energies of the core chemical components and core targets of XJZT were both <−5.0 kcal·mol^−1^, and molecular docking diagrams of the top 3 core target genes and chemical components were displayed (Fig. 7).

### 3.6. DEG identification results

**Figure 8.**
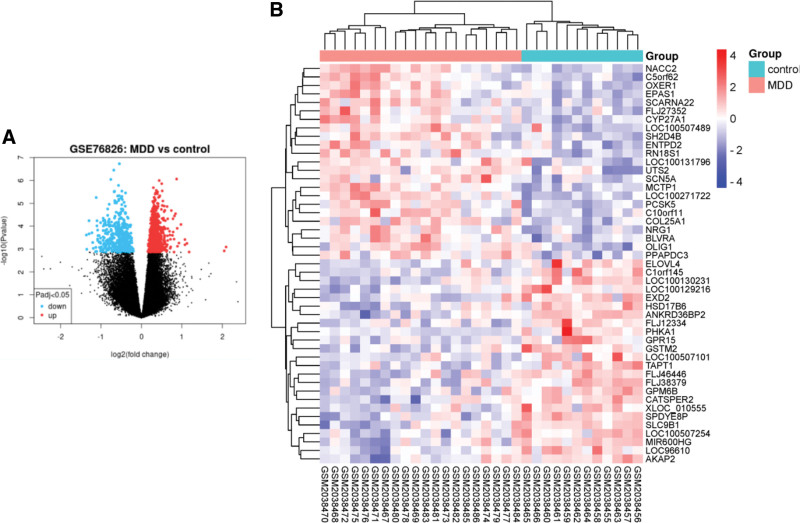
DEG analysis. (A) The volcano map of DEG. The red point represents the upregulated DEG, while the blue point represents the downregulated DEG. (B) The hot map of DEG. According to logFC, the top 50 DEG are labeled in the graph. DEG = differentially expressed gene.

To verify whether the core target genes of XJZT treatment for MDD also undergo changes in the patient’s body, we selected the blood transcriptome dataset of MDD patients for differential gene analysis. Our research results showed that we obtained a total of 1343 DEGs in the GSE76826 dataset, of which 936 were downregulated and 407 were upregulated (Fig. 8).

### 3.7. WGCNA results

**Figure 9.**
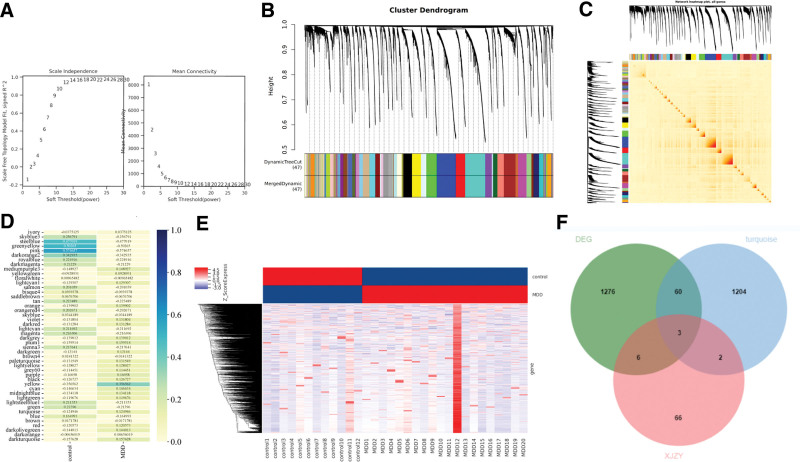
WGCNA analysis. (A) The higher the correlation coefficient (maximum of 1), the closer the network is to the scale-free network distribution, the power value chosen for this study is 10. (B) The upper part of the figure is a gene clustering tree constructed from the disTOM matrix of weighted correlation coefficients; the lower part of the figure shows the distribution of genes in each module, with the same color representing the same module, and the turquoise module contains the most genes. (C) All gene clustering heatmaps. (D) The correlation heatmap of trait modules shows that the correlation coefficient of turquoise module in the MDD group is 0.124946, while in the control group it is −0.124946. (E) Based on the division results of the WGCNA module, use clustering heatmaps to display the gene expression information of the turquoise module. (F) Venn diagram of DEG, turquoise module genes and target genes for XJZT treatment of MDD. DEG = differentially expressed gene, MDD = major depressive disorder, WGCNA = weighted gene co-expression network analysis, XJZT = Xiaojian Zhongtang.

The WGCNA analysis results of the GSE76826 dataset show that the clustering effect of the samples is great, and there are no outliers. The first value to reach the scale-free topological fitting index (0.8) is 10, so when the soft threshold is 10, the value starts to remain stable, indicating good network connectivity (Fig. 9A). GSE76826 consensus identifies 46 modules, among which the turquoise module contains 1269 genes, which is the largest gene module (Fig. 9B–E). To verify whether the core target genes of XJZT for MDD treatment have changed in patients, we uploaded turquoise module genes, DEG, and potential target genes of XJZT treatment for MDD to the Venny 2.1.0 platform, and obtained 3 intersecting genes, namely AKT1, D(4) dopamine receptor (DRD4), and kynurenine 3-monooxygenase (KMO) (Fig. 9F).

### 3.8. GSEA results

**Figure 10.**
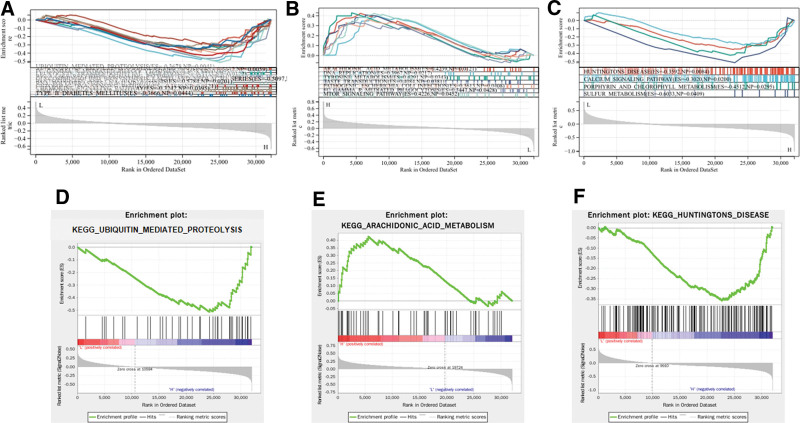
GSEA analysis. (A) Twelve pathways were obtained after AKT1 enrichment. (B) Seven pathways were obtained after DRD4 enrichment. (B) Four pathways were obtained after KMO enrichment. (D) Among the enriched pathways of AKT1, ubiquitin-mediated proteolysis is the most significant. (E) Among the enriched pathways of DRD4, arachidonic acid metabolism is the most significant. (F) Among the enriched pathways of KMO, Huntington disease is the most significant. AKTI = RAC-alpha serine/threonine-protein kinase, DRD4 = D(4) dopamine receptor, GSEA = gene set enrichment analysis, KMO, kynurenine 3-monooxygenase.

To determine the gene functions and pathways of AKT1, DRD4, and KMO, we conducted GSEA analysis on these 3 genes. The results showed that AKT1 mainly involves 12 pathways, with the most significant being ubiquitin-mediated proteolysis (Fig. 10A and D); DRD4 mainly involves 7 pathways, with the most significant being AA metabolism (Fig. 10B and E); KMO mainly involves 4 pathways, with the most significant being Huntington disease (Fig. 10C and F).

### 3.9. ROC analysis results

**Figure 11.**
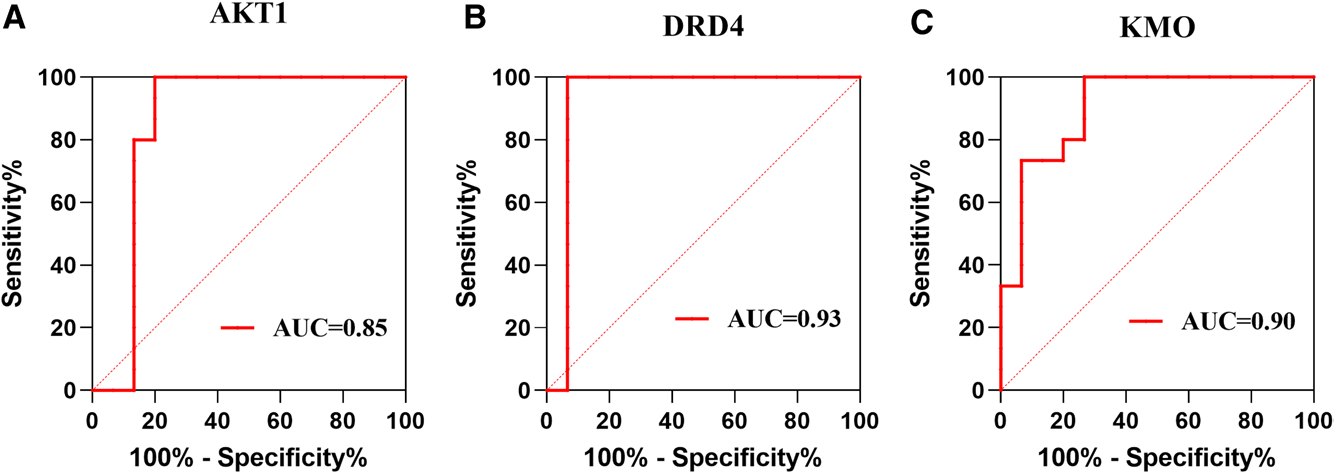
ROC analysis. (A) ROC curve of AKT1, AUC = 0.85, (B) ROC curve of DRD4, AUC = 0.93, (C) ROC curve of KMO, AUC = 0.90. AKT1 = RAC-alpha serine/threonine-protein kinase, AUC = area under ROC curve, DRD4 = D(4) dopamine receptor, KMO = kynurenine 3-monooxygenase.

Due to the occurrence of MDD in the brain, in order to verify whether AKT1, DRD4, and KMO have also changed in the patient’s brain tissue, we chose the transcriptome dataset GSE54568 of MDD brain tissue for ROC analysis. The results showed that changes in AKT1, DRD4, and KMO were indeed found in the GSE54568 dataset, with AUC values of 0.85 for AKT1, 0.93 for DRD4, and 0.90 for KMO (Fig. 10A–C). This indicates that AKT1, DRD4, and KMO can serve as diagnostic markers for MDD and are also the core target genes for XJZT treatment of MDD (Fig. 11).

## 4. Discussion

The concept of MDD can be traced back to 1780, when symptoms were mainly characterized by low mood and were not treated as an independent disease. It was not until the late 18th century that a modern depression diagnosis and treatment system was established.^[22]^ The traditional Chinese medicine compound XJZT was first used to treat symptoms of irritability and anxiety after catching a cold, and has the effect of enhancing digestive function. Traditional Chinese medicine theory suggests that the spleen is related to emotional depression, and enhancing the function of the spleen can help combat depression. However, the specific pathway for XJZT to treat MDD is not yet clear. Network pharmacology is an emerging system biology approach that can connect the chemical components of plant drugs with disease targets, and conduct gene function analysis based on various bioinformatics databases to clarify the therapeutic mechanisms of chemical components in natural products for diseases.^[23]^ Bioinformatics can identify the molecular differences between patients and healthy individuals from multilevel microarray data, and is an effective research method for exploring the potential molecular mechanisms of diseases.^[24]^ Therefore, in this study, we integrated network pharmacology and bioinformatics to explore the core chemical components and target genes of XJZT in treating MDD, and validated them based on transcriptome datasets of blood and brain tissues from MDD patients in the GEO database to clarify the specific molecular mechanism of XJZT in treating MDD.

Through network pharmacology, we have identified the top 10 core chemical components of XJZT for the treatment of MDD, including quercetin, beta-sitosterol, maltose, stigmasterol, kaempferol, medicarpin, nuciferin, steroline, isorhamnetin, and stepharine. Beta-sitosterol and stigmasterol are cholesterol compounds that are widely present in the leaves, roots, and fruits of various plants in nature. They have various pharmacological activities such as anti-inflammatory, antioxidant, cholesterol-lowering, blood glucose lowering, and immune regulation.^[25,26]^ Metabolomics studies have shown that abnormal changes in lipid metabolism, energy metabolism, and glucose metabolism were found in the plasma and brain of chronic unpredictable mild stress model rats. After intervention with herbs containing beta-sitosterol and stigmasterol, the depressive symptoms of chronic unpredictable mild stress model rats were significantly improved, and these components did not have any damaging effects on liver function.^[27,28]^ Quercetin is the most common polyphenolic flavonoid compound among the most natural products, found in various fruits, vegetables, and plant medicines, with anti-inflammatory and antioxidant activities.^[29,30]^ Studies have shown that quercetin can significantly prolong the sleep time induced by thiopental sodium in mice and exhibit a dose-dependent sedative effect. In addition, inflammatory factors in the brain of lipopolysaccharide-induced depression model rats are significantly increased, but they are significantly reduced after quercetin intervention.^[31,32]^ Maltose is the main hydrolysis product of macromolecular polysaccharides such as starch, glycogen, and dextrin under the catalysis of β-amylase. Traditional Chinese medicine theory suggests that maltose has the function of nourishing the spleen and lungs. Studies have found that maltose can increase the expression level of BDNF in the plasma of MDD patients and enhance their cognitive and memory functions.^[33]^ Flavonoids are natural antioxidants, and kaempferol is the most abundant alkyl flavonoid in plant medicine. Kaempferol can alleviate depressive-like behavior in chronic social defeat stress model mice by regulating the AKT/β-catenin signaling pathway, increase the content of superoxide dismutase, catalase, glutathione peroxidase, interleukin-1.^[34]^ Selective D1 receptor (D1R) agonists have strong antidepressant like effects, while tetrahydroberberine extracted from the herbal *Stephania intermedia* has strong affinity for both D1R and D2R, and promotes dopamine release.^[35]^ Research has shown that stepholidine can alleviate depressive behavior in chronic mild stress model rats by activating the D1R-mediated cAMP/PKA/mTOR pathway and enhancing long-term potentiation.^[36]^

Through PPI analysis, we identified the top 10 core target genes for XJZT treatment of MDD, including AKT1, albumin, IL-6, TNF, IL-1β, TP53, EGFR, protein c-fos, EGF, and PTGS2. Numerous studies have shown that cytokines and their driven signaling pathways are associated with depression. In MDD, cytokines from peripheral blood are transported to effector cells, which are then transported to the brain and interfere with the release of neurotransmitters.^[37]^ IL-1β, IL-6, and TNF are classic inflammatory factors that have been shown to promote the progression of MDD.^[38]^ Studies have shown that IL-1β, IL-6, and TNF are significantly elevated in the plasma and cerebrospinal fluid of MDD patients, and the content of these inflammatory factors is positively correlated with disease severity.^[39]^ Another study found that the expression level of TP53 was significantly increased in the plasma transcriptome of MDD patients.^[40]^ EGF can bind to EGFR, promoting cell growth, proliferation, and differentiation. EGF and EGFR can be detected in nerve cells of manic patients.^[41]^ Studies have shown that in the plasma of adolescent patients with MDD, EGF and EGFR are elevated, while BDNF is significantly decreased, suggesting that EGF and EGFR may promote the occurrence of MDD.^[42]^ PTGS2, also known as cyclooxygenases 2, is the first key rate-limiting enzyme that catalyzes AA and prostaglandin synthesis. It is activated by EGF and promotes the activation of inflammatory cytokines in cells.^[43]^ In MDD, PTGS2 can inhibit the expression of 5-HT and promote the elevation of IL-1β, IL-6, and TNF, which is significantly reversed after administration of PTGS2 inhibitors.^[43]^ Through WGCNA and GSEA, we further identified 3 core genes for XJZT treatment of MDD, including AKT1, D(4) DRD4, and KMO, which mainly involve ubiquitin-mediated proteolysis, AA metabolism, and Huntington disease. The subtype gene AKT1 of the protein kinase B is highly correlated with the occurrence of MDD. Studies have shown that there is a significant positive correlation between AKT1 rs2494746 and rs3001371 polymorphisms in the plasma of 461 Chinese MDD patients with suicidal and anxiety tendencies,^[44]^ and a decrease in AKT1 was detected in the brain of MDD patients who committed suicide, while glycogen synthase kinase 3β rise.^[45]^ We found through GSEA that AKT1 is enriched in the ubiquitin-mediated proteolysis pathway, while activation of the PI3K/AKT1/mTOR pathway activates autophagy, promoting rapid degradation of neurons damaged by inflammatory factors in the hippocampus of MDD model rats, restoring synaptic function, and thus exerting neuroprotective effects.^[46,47]^ The DRD4 gene is located near the telomere of chromosome 11p and exhibits a higher number of polymorphisms.^[48]^ Compared to other types of genes, the complex polymorphism of the DRD4 gene has been shown to be associated with abnormal mental behavior, such as attention deficit hyperactivity disorder, mania, and MDD.^[49]^ DRD4 is enriched in the AA metabolism pathway. Studies have shown that in the plasma of 137 patients with recurrent MDD, eicosapentaenoic acid, docosahexaenoic acid, and AA concentrations decreased, and nocturnal cortisol concentrations increased, indicating that abnormal changes in AA can lead to hypothalamic pituitary adrenal axis disorder.^[50]^ KMO is an intermediate product of TRP breakdown and metabolism by canine uric acid.^[51]^ TRP is catalyzed by indoleamine-2,3-dioxygenase, and tryptophan 2,3-dioxygenase to produce N-formyl-L-kynurenine, which is then rapidly converted to L-kynurenine (KYN), and KMO is one of the 3 metabolites of KYN.^[52]^ KMO can produce toxic metabolites and mediate the occurrence of various diseases, such as Huntington disease, Parkinson disease, Alzheimer disease, and the most common MDD.^[53]^ Studies have shown that high levels of IL-6, IL-2, and TNF are present in the plasma of MDD patients-α It can enhance the activity of indoleamine-2,3-dioxygenase and KMO, not only consuming TRP, but also promoting KYN metabolism to generate excessive 3-hydroxykynurenine and quinolinate.^[54]^

## 5. Conclusion

In this study, we identified that the main components of XJZT in the treatment of MDD include quercetin, beta-sitosterol, maltose, kaempferol, naringenin, and stepholidine by integrating network pharmacology and bioinformatics techniques. These core components can regulate key targets of MDD, including AKT1, KMO, and DRD4. Their potential molecular mechanisms may be related to the regulation of ubiquitin-mediated proteolysis, AA metabolism, and Huntington disease pathways. In conclusion, this study provides a reference for the intervention mechanisms of the traditional Chinese medicine formula XJZT in treating MDD, which may aid in identifying early diagnostic markers for MDD and in the development of targeted drugs.

## Author contributions

**Writing—original draft:** Huaning Jiang, Jian Zhang.

**Writing—review & editing:** Quan Li, Yanyan Zhou.
